# Characterization and Construction of Full-Length cDNA Infectious Clone of a Novel BCMV Isolate in Pathogroup III

**DOI:** 10.3390/plants14213359

**Published:** 2025-11-02

**Authors:** Jinglei Zhang, Li Dong, Jue Zhou, Sifan Huo, Haixu Feng, Chenchen Jing, Xue Feng

**Affiliations:** Shanxi Key Laboratory of Integrated Pest Management in Agriculture, College of Plant Protection, Shanxi Agricultural University, Taiyuan 030031, China; zhangjinglei0514@163.com (J.Z.); m15163152497@163.com (L.D.); m19858002636@163.com (J.Z.); hwslrbsyyj@163.com (S.H.); fenghaixu007@163.com (H.F.)

**Keywords:** *Bean common mosaic virus*, infectious cDNA clone, pathogenicity

## Abstract

*Bean common mosaic virus* (BCMV; *Potyvirus phaseovulgaris*) is one of the primary viruses that severely impacts the yield and quality of common beans (*Phaseolus vulgaris* L.) and has a worldwide distribution. Utilizing small RNA sequencing and RT-PCR validation, this study identified widespread co-infection by multiple viruses in field-collected common bean samples, with BCMV being the dominant viral species. A novel isolate, designated DY9, was obtained from these field samples. Pathotype characterization confirmed DY9 as pathotype PG-III, while previous studies reported all other PG-III members as *Bean common mosaic necrosis virus* (BCMNV). Whole-genome sequencing and phylogenetic analysis revealed that DY9 was genetically closer to BCMV and diverged significantly from known PG-III isolates. Based on these findings, we constructed an infectious clone of DY9. To address the genetic instability of *Potyvirus* in the *Escherichia coli* (*E. coli*) expression system, we discovered that inserting Intron 2 (derived from the *NiR* gene of *P. vulgaris*, GenBank: U10419.1) at position 2431 of the HC-Pro gene and targeting Intron 1 (derived from the *ST LS1* gene of *Solanum tuberosum*, GenBank: X04753.1) at position 4240 of the CI gene significantly improved the stability of the cloning vector. The clone was verified to systemically infect common bean plants and induce typical mosaic symptoms. Infectivity was validated through RT-PCR, RT-qPCR, Western blotting, and transmission electron microscopy. This study represents the first successful construction of an infectious clone for pathotype PG-III BCMV, providing a critical reverse genetics tool for dissecting viral pathogenesis and identifying resistance genes. These findings not only expand the genetic diversity of BCMV but also offer a methodological reference for constructing infectious clones of *Potyvirus* species.

## 1. Introduction

*Bean common mosaic virus* (BCMV; *Potyvirus phaseovulgaris*) is a member of the genus *Potyvirus* within the family *Potyviridae* [1]. In 1917, Stewart and Reddick isolated the virus responsible for mosaic disease in bean plants [2]; in 1934, Pierce formally named the virus BCMV [3]. Since its discovery, BCMV has been extensively documented across diverse geographic regions worldwide [4,5,6]. In China, outbreaks of BCMV have been reported in major common bean-producing provinces, including Zhejiang, Yunnan, Liaoning, and Heilongjiang [7,8,9]. BCMV spreads through various means, primarily via seeds, mechanical inoculation and a range of aphid species in a non-persistent manner [10,11,12]. It possesses a wide host range and is among the most damaging viruses that naturally infect leguminous plants. Its primary host is the common bean (*Phaseolus vulgaris* L.). BCMV infection in beans can induce symptoms such as mosaic patterns, malformation, yellowing, and stunting. In severe cases, it can lead to local or systemic necrosis, ultimately resulting in plant death [13,14].

BCMV exhibits high genetic variability and frequent intra- and interspecific recombination [15], resulting in a multitude of strains. To date, numerous BCMV isolates from diverse geographic regions have been reported globally, and complete genomic sequences for many of these isolates have been obtained. However, a unified classification system for BCMV strains does not currently exist on a global scale. Various methods, including the assessment of biological characteristics on differential hosts, serological reactions, reversed-phase high-pressure liquid chromatography (HPLC) peptide mapping, and molecular biology techniques, have been employed to differentiate between BCMV strains [4]. For pathotype classification, BCMV isolates are categorized into eight pathotypes (PG I–PG VIII) based on their differential reactions to 12–14 common bean cultivars that carry defined resistance gene combinations [16]. Pathotypes PG I–PG VII were initially proposed by Drijfhout et al. [17], while PG-VIII was later identified by Feng et al. [18]. Among these, with the exception of the previously reported RU1 isolate, which has been classified as belonging to pathogenicity group PG-VI of BCMV strains [19,20,21], the primary members of pathogenicity groups PG-III and PG-VI are the Bean common mosaic necrosis virus (BCMNV) [22], a closely related virus within the same genus [23].

Full-length viral cDNA infectious clones are essential tools in reverse genetics for plant virus research [24]. Researchers have successfully constructed infectious clones for numerous highly pathogenic viruses, which are widely utilized in studies such as the analysis of pathogenic mechanisms and the screening of disease-resistant varieties [25,26]. However, constructing infectious clones for viruses in the genus *Potyvirus* presents significant challenges due to their large genome size (approximately 10 kb), structural complexity, and the presence of prokaryotic promoter-like elements within the viral genome [27,28]. During prokaryotic amplification, these elements drive cytotoxic product expression, impairing host growth [29,30]. Consequently, the viral sequence is highly susceptible to recombination with sequences from the prokaryotic host or other exogenous genes. This often results in plasmid instability or loss of clone infectivity, thereby substantially increasing the difficulty of constructing infectious clones. To address these challenges, researchers have explored various strategies, including the use of low-copy vectors [31], targeted intron insertion [28,30,32], yeast homologous recombination [26,33,34], and in vitro ligation prior to inoculation [35].

Through small RNA sequencing and RT-PCR validation, this study confirmed that BCMV is the predominant viral population infecting field-grown leguminous crops. The DY9 strain was successfully isolated from symptomatic field plants and characterized as a BCMV isolate belonging to pathotype PG-III through a combination of pathotypic analysis and phylogenetic profiling. Based on these findings, this study successfully constructed an infectious clone of the isolated strain by introducing introns at the 2431 position of the *HC-Pro* gene and the 4240 position of the *CI* gene, further confirming its infectivity. This clone provides a critical reverse genetics tool for studying BCMV pathogenesis mechanisms and expanding knowledge of viral genetic diversity.

## 2. Results

### 2.1. Small RNA Sequencing Results and Isolation of BCMV Strains

The collected four types of legumes were grouped by their four sample codes to form composite samples, resulting in a total of five composite samples. Following small RNA sequencing, the results were presented in Table 1. Diseased leaf samples yielded 96,526,571 raw reads; after quality trimming, 88,008,021 high-quality reads (18–35 nt) remained, comprising 91.17% of total small RNA reads.

**Figure 1:**
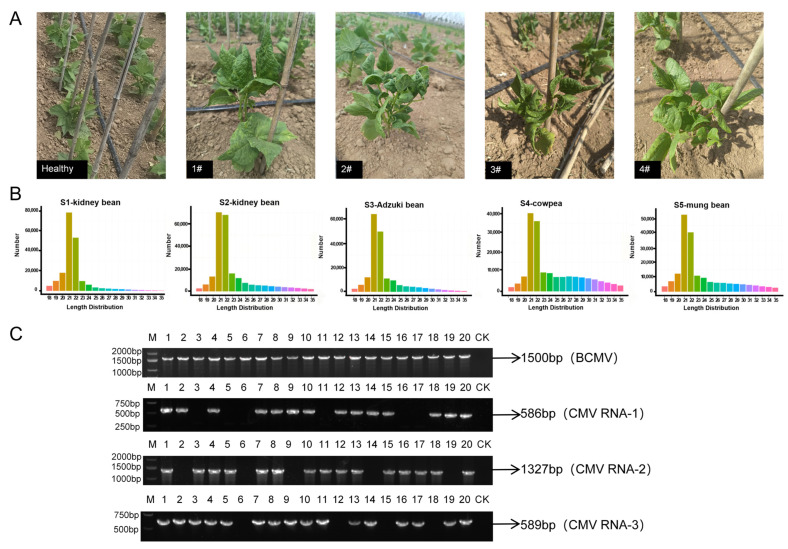
Small RNA sequencing and RT-PCR validation of BCMV infection in common bean plants. (**A**) Field photographs of legumes. The panel shows a healthy plant (left) and four plants (1–4) displaying symptoms suspected to be caused by viral infection. (**B**) Length distribution profile of all clean reads derived from high-throughput small interfering RNA (siRNA) deep sequencing of five pooled samples. Columns of different colors represent reads of varying lengths (15–34 nt). (**C**) RT-PCR confirmation of viral presence in individual samples. Lane M: 1 kb DNA marker; Lanes 1–20: 20 samples of infected legumes; Lane CK: healthy control sample.

To identify the viral species, we first assembled sRNA reads using Velvet software (v1.0) with a k-mer value of 17. The resulting contig sequences were aligned against viral databases (Table 2), yielding major matches with effective contig sequences of BCMV and *Cucumber Mosaic Virus* (CMV). To more precisely quantify the relative abundance of these two viruses, we further directly aligned all high-quality reads against the reference genomes of BCMV and CMV. Quantitative analysis revealed that across five pooled samples, a total of 1,971,380 reads specifically aligned to the BCMV genome, accounting for 2.24% of all high-quality reads. In contrast, 1,126,502 reads aligned to the CMV genome, representing 1.28% of the total. We then analyzed the characteristics of the entire sRNA sequencing library. The length distribution of all high-quality clean reads showed that 21-nt small RNAs were the most abundant class (Figure 1B). The sRNA length distribution reflected reliable sequencing and assembly quality. These results demonstrate that the abundance of BCMV is substantially higher than that of CMV. This conclusion is supported by both the initial identification based on effective contigs (Table 2) and, more definitively, by the precise quantification of virus-specific reads. This strongly indicates that BCMV was the predominant viral population in the legume crops surveyed in this field study.

To further validate small RNA sequencing results and characterize viral infections at the plant level, we designed gene-specific primers targeting conserved regions of BCMV and CMV contigs and used them for the RT-PCR analysis of individual samples. The detection results showed that 19 out of 20 samples were co-infected with BCMV and CMV (Figure 1C), while only the DY9 sample tested negative for CMV. Additional testing of the DY9 sample for other common field viruses, including *Soybean mosaic virus* (SMV), *Bean yellow mosaic virus* (BYMV), and *Cowpea aphid-borne mosaic virus* (CABMV), also yielded negative results. This confirmed that the DY9 sample was exclusively infected by BCMV. However, we were unable to directly associate BCMV infection alone with the observed symptoms, as the mixed infections in these samples made it difficult to distinguish virus-specific symptom patterns. Accordingly, this sample was designated BCMV-DY9 and maintained in *Nicotiana benthamiana* (*N. benthamiana*) for subsequent experiments.

### 2.2. BCMV-DY9 Sequencing and Phylogenetic Analysis

To obtain the complete viral genome sequence, BCMV-specific primers were designed to amplify nine overlapping fragments. The full genome sequence of DY9, including the 5′ and 3′ untranslated regions (UTRs), was assembled using SeqMan software, resulting in a genome of 10,022 nucleotides (nt) that encodes 3319 amino acids (aa) (Additional File S2). Following whole-genome sequencing, BLAST (v2.16.0) analyses against the NCBI database identified DY9 as sharing the highest nucleotide identities with referenced BCMV isolates across all genomic regions: 5′-UTR (93.75%), 3′-UTR (94.58%), *P1* (94.64%), *HC-Pro* (92.34%), *P3* (93.09%), *P3N-PIPO* (97.84%), *6K1* (94.23%), *CI* (93.11%), *6K2* (93.71%), *NIa-VPg* (88.75%), *NIa-Pro* (94.65%), *NIb* (94.96%), and *CP* (99.42%). The overall nucleotide sequence identity of the DY9 genome to other BCMV isolates ranged from 85.40% to 91.61%, while its identity to BCMNV isolates ranged from 75% to 80%. The nucleotide sequence identity of the DY9 *CP* gene to BCMV isolates ranged from 90% to 99%, and to BCMNV isolates from 75% to 80%. Based on the sequence identity analysis (Table 3), DY9 exhibited the highest nucleotide identity to the Chinese BCMV isolates 22Huhe (GenBank: OR778613.1, 91.61%) and HY (GenBank: LC582403.1, 90.57%), followed closely by the Tanzanian isolate BCMV-HXH-2 (GenBank: MF405191.1, 90.56%).

**Figure 2:**
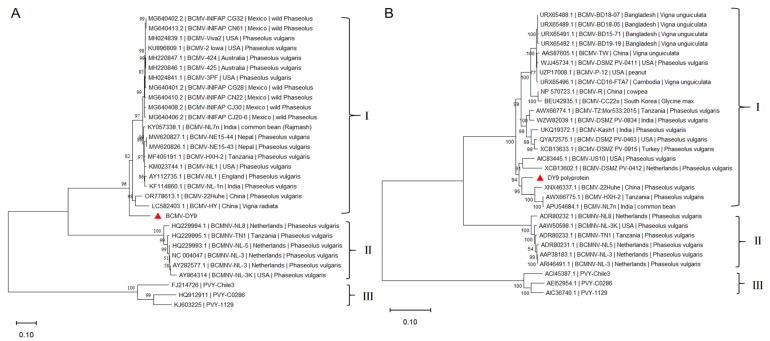
Phylogenetic analysis of BCMV strains and related viruses based on complete genomic nucleotide and amino acid sequences. (**A**) Neighbor-Joining phylogenetic tree constructed using nucleotide sequences. (**B**) Neighbor-Joining phylogenetic tree constructed using amino acid sequences. Note: The whole genome sequences of BCMV were downloaded from NCBI, with BCMNV and PVY included as outgroups. Phylogenetic analysis was performed using MEGA11 software. “▲”: isolates obtained in this study. Scale represented genetic distance. Numbers at the nodes indicate bootstrap support percentages.

To infer evolutionary relationships among DY9 and related viruses, we performed phylogenetic analyses using aligned nucleotide and amino acid sequences from 20 BCMV, 6 BCMNV, and 3 *Potato virus Y* (PVY) isolates. The Neighbor-Joining (NJ) phylogenetic tree demonstrated high robustness, with bootstrap values of ≥70% at most nodes (Figure 2). The results indicated that all 30 isolates clustered into three distinct clades at both the nucleotide and amino acid levels: BCMNV and PVY formed monophyletic clades, while DY9 grouped with other BCMV isolates, displaying the closest phylogenetic relationship and the highest nucleotide sequence identity. Phylogenetic analysis based on the amino acid sequence of BCMV-DY9 further revealed that it clustered most closely with the Chinese isolate BCMV-22Huhe (GenBank: XNX46337.1), the Tanzanian isolate BCMV-HXH-2 (GenBank: AWX66775.1), and the Indian isolate BCMV-NL7n (GenBank: APU54684.1). As illustrated in the phylogenetic tree (Figure 2), the majority of BCMV isolates had *P. vulgaris* as their host. This finding suggests that BCMV isolates infecting common beans exhibit a close phylogenetic relationship, although no clear geographical correlation was observed.

### 2.3. Identification of BCMV-DY9 Pathotypes

When screened across 11 cultivars of *P. vulgaris* with diverse genetic backgrounds, wild-type BCMV-DY9 isolate induced typical mosaic mottling, leaf malformation, and stunted growth in susceptible cultivars (DW, SGR, Sanilac). The presence of BCMV in the inoculated plants was confirmed by RT-PCR, with results consistent with biological assays. Based on the pathogenicity spectrum revealed by the variations in resistance among the bean cultivars, wild-type BCMV-DY9 isolate was classified as PG-III (Table 4).

### 2.4. Construction of pBCMV-DY9 Clone and Determination of Biological Activity

To better understand the biological characteristics of the BCMV DY9 strain, we tried to construct its infectious clone. We initially inserted a single intron into the *CI* (position 4240) and two introns into *P3* (position 2955) and *CI* (position 4240), which is a common insertion site for potyviruses, to create two clones: pBCMV-DY9-1 and pBCMV-DY9-2. Sequencing analysis of pBCMV-DY9-1 showed numerous mutations throughout the plasmid, with a high density observed in the *HC-Pro* region. In contrast, although the pBCMV-DY9-2 construct (containing two introns) yielded a stable, mutation-free recombinant plasmid, it failed to exhibit infectious capability. Guided by the sequencing findings from pBCMV-DY9-1, we strategically inserted introns into the AG-GT motifs of the *HC-Pro* gene at position 2431 and the *CI* gene at position 4240. The resulting clone, pBCMV-DY9-3 (hereafter uniformly referred to as pBCMV-DY9), was validated through segmental amplification using nine pairs of specific primers. Subsequent full-length genome sequencing confirmed that the sequence of this clone was identical to that of the wild-type virus DY9 (WT-DY9) control, including the intron sequences, with no mutations detected (Additional File S2).

**Figure 3:**
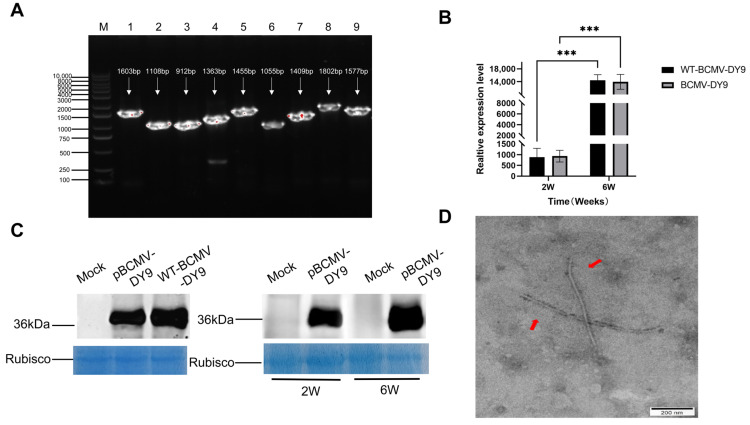
Biological activity of BCMV clones (pBCMV-DY9) and wild-type virus DY9 (WT-DY9) in *Nicotiana benthamiana* (*N. benthamiana*). (**A**) RT-PCR detection of pBCMV-DY9 in systemic leaves of *N. benthamiana* at 2 weeks post-inoculation (wpi). Detection was performed using the same 9 pairs of specific primers as those used for full-length genome sequencing of BCMV-DY9, covering the full-length genome of pBCMV-DY9; different bands correspond to amplified products of distinct genomic regions. (**B**) Relative expression of BCMV CP gene in *N. benthamiana* infected with pBCMV-DY9 and WT-DY9, measured by qPCR at 2 and 6 wpi. Data are presented as mean ± SEM (*n* = 3). Statistical significance was determined by two-way ANOVA followed by Šídák’s multiple comparisons test (*** *p* < 0.001). (**C**) Western blot analysis of BCMV CP accumulation in *N. benthamiana* using BCMV CP-specific antibodies. Left panel: comparative accumulation of WT-DY9 and pBCMV-DY9 CP proteins at 2 wpi; right panel: accumulation of pBCMV-DY9 CP proteins at 2 and 6 wpi. Mock: negative control plants inoculated with the pCB301 empty vector. The Coomassie blue-stained Rubisco large subunit (Rub L) is shown as a loading control. (**D**) The viral particles observed in a systemic leaf of *N. benthamiana* infected with pBCMV-DY9 via TEM. Scale bars, 200 nm.

A stable, mutation-free *Escherichia coli* (*E. coli*) plasmid was introduced into *Agrobacterium tumefaciens* (*A. tumefaciens*). Subsequently, the recombinant plasmid pBCMV-DY9 was delivered into *N. benthamiana* via *Agrobacterium*-mediated infiltration (Agroinfiltration). Healthy *N. benthamiana* plants infiltrated with the empty vector pCB301 served as the negative control. At two weeks post-agroinfiltration, *N. benthamiana* plants exhibited no discernible symptoms of viral infection, consistent with the characteristic phenotype induced by conventional BCMV in this host. Total RNA extracted from systemic leaves was subjected to RT-PCR analysis (Figure 3A). To verify the integrity of the pBCMV-DY9 genome, this detection employed 9 pairs of specific primers that cover the full-length genome of pBCMV-DY9, with each pair targeting a distinct region of the genome. Consequently, the amplified products showed differences in size. The presence of these target bands confirmed that the pBCMV-DY9 infectious clone possesses biological activity—it can establish effective infection in tobacco plants and initiate the viral replication cycle, laying the foundation for subsequent functional validation. Further validation using RT-qPCR demonstrated that the clone replicated efficiently in systemic leaves, with viral accumulation levels comparable to those of the wild-type DY9 virus. Viral transcript abundance rose significantly between 2 and 6 weeks post-inoculation (wpi), as depicted in Figure 3B.

Western blotting of systemic leaves from pBCMV-DY9-infected *N. benthamiana* detected a ~36 kDa band corresponding to the coat protein (CP) and consistent with the wild-type virus. Viral protein abundance rose significantly between 2 and 6 wpi (Figure 3C), corroborating quantitative RT-qPCR findings. Additionally, viral particles were crudely purified from *N. benthamiana* inoculated with pBCMV-DY9 via agroinfiltration, and the viral particles of the infectious clone were observed under a transmission electron microscope (TEM) using the negative staining method. TEM revealed that pBCMV-DY9 exhibits a typical curved filamentous viral particle morphology, further confirming the normal expression of pBCMV-DY9 viral particles (Figure 3D). Notably, this clone retained genomic stability while supporting robust viral protein expression in *N. benthamiana*. Collectively, these data confirm that pBCMV-DY9 constitutes a biologically functional infectious clone.

**Figure 4:**
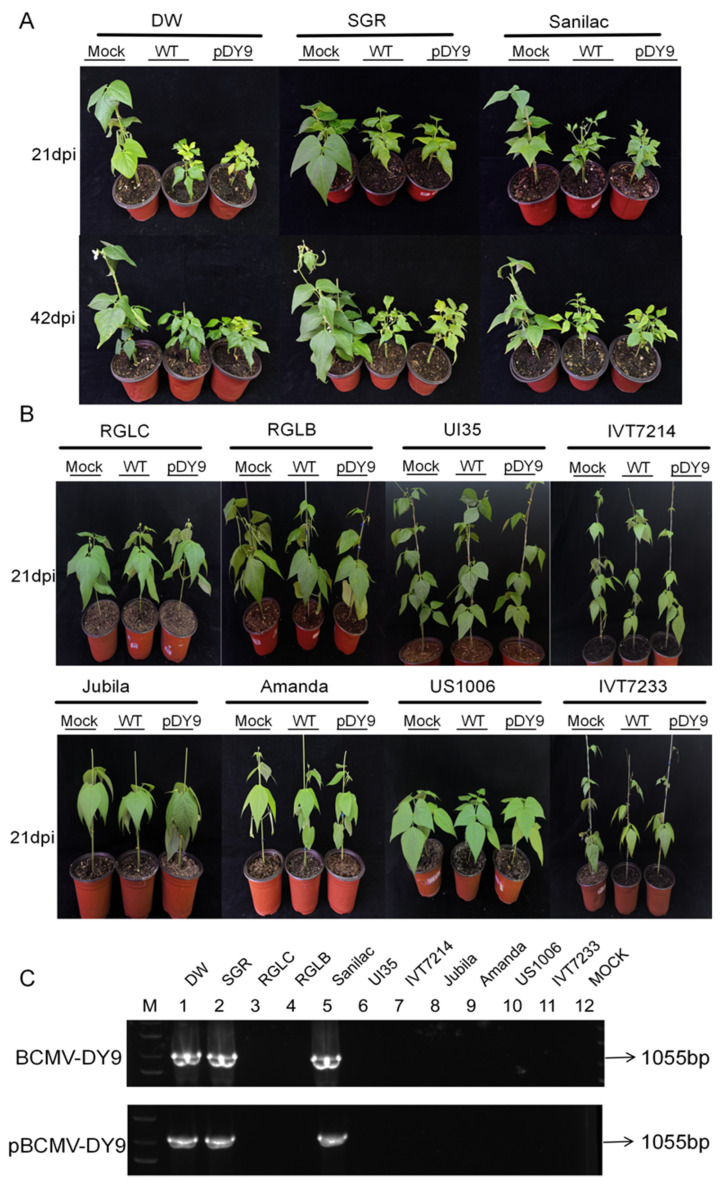
Pathogenicity assessment and RT-PCR validation of BCMV-DY9 infection in common beans. (**A**) Symptom map of BCMV-inoculated infected plants at 21 and 42 days post-inoculation (dpi). (**B**) Symptom map of uninfected plants inoculated with BCMV at 21 dpi. From left to right: plants inoculated with 1× PBS (mock), WT-DY9, and recombinant pBCMV-DY9 virus (pDY9). (**C**) RT-PCR confirmation of viral replication in 11 diverse cultivars infected with WT-DY9 or pBCMV-DY9 at 21 dpi. Lane M: 1 kb DNA marker; Lanes 1–11: individual cultivars; Lane 12: negative control.

To verify the biological activity of progeny viruses generated by the infectious cDNA clone, a virus inoculum was mechanically rub-inoculated onto 11 common bean cultivars with varying genetic backgrounds (Figure 4). At 21 days post-inoculation (dpi), cultivars DW, SGR, and Sanilac exhibited characteristic BCMV symptoms—mosaic patterning, chlorosis (yellowing), leaf deformation, and stunted growth. By 42 dpi, the infected plants exhibited growth retardation, downward curling and deformation of the diseased leaves, and produced few or almost no pods, which were consistent with the symptoms caused by the wild-type DY9. The remaining common bean cultivars displayed no obvious symptoms, aligning with the biological phenotype of healthy controls. RT-PCR analysis using primers DY9-6F/6R specifically amplified a 1055 bp fragment in symptomatic tissues (Figure 4C), thereby corroborating the observed phenotypes. These findings demonstrate that pBCMV-DY9 retains full pathogenicity comparable to wild-type BCMV and maintains unimpaired infectivity.

## 3. Discussion

In recent years, the rapid advancement of high-throughput sequencing technology has led to its increasingly widespread application in the detection of unknown plant viruses [36]. In this study, small RNA deep sequencing technology was utilized to perform small RNA sequencing on a cDNA library created by combining common beans, adzuki beans, cowpeas, and mung beans. A total of five viruses were identified, with notable instances of mixed infections. These viruses, ranked by detection rate from highest to lowest, included BCMV, CMV, SMV, BYMV, and CABMV. Among these, BCMV and CMV were the predominant viral species, exhibiting significantly higher detection rates compared to the other viruses.

To gain deeper insights into the biological characteristics and pathogenic mechanisms of BCMV, this study systematically combined field investigations with laboratory research. Through field sample collection and virus isolation techniques, a viral isolate was successfully obtained and designated as DY9. Pathotype identification revealed that this isolate belongs to the PG-III pathotype. Since all known strains in the current PG-III classification are categorized as BCMNV, the primary objective of this study was to clarify the taxonomic affiliation of this isolate (BCMNV or BCMV). According to the classification criteria for *Potyviruses* outlined in the 9th Report of the International Committee on Taxonomy of Viruses (ICTV), viruses can be considered distinct species if the amino acid sequence similarity of their large open reading frame (ORF) or coat protein (*CP*) gene is less than 82%, or if the nucleotide sequence similarity is less than 76% [37]. To address this, whole-genome nucleotide sequence alignment revealed that the DY9 isolate shares 85.40% to 91.61% identity with BCMV isolates and 75% to 80% identity with BCMNV isolates. Similarly, its *CP* gene nucleotide sequence shares 90% to 99% identity with BCMV isolates and 75% to 80% identity with BCMNV isolates. Concurrently, phylogenetic analysis demonstrated that BCMNV members form a distinct monophyletic clade, while DY9 clusters closely within the evolutionary clade formed by typical BCMV isolates, indicating a closer phylogenetic relationship. Collectively, based on both pathogenicity classification and molecular biological characteristics, these findings confirm that DY9 is a BCMV isolate belonging to pathogroup PG-III.

Despite substantial advances in viral infectious cloning technologies, the development of a universal cloning strategy remains a critical technical challenge in this field. For viruses of the genus *Potyvirus*, which possess complex genomic structures, the construction of infectious clones presents dual technical challenges. On the one hand, the genomic length of approximately 10 kb significantly complicates cloning operations; on the other hand, the expression of toxic proteins driven by cryptic promoters within the genome leads to genetic instability of clone intermediates in the *E. coli* system. To mitigate this latter issue, intron insertion has emerged as an effective strategy to suppress the abnormal expression of viral toxic proteins in bacterial hosts. Previous studies have indicated that the genomic instability of *Potyvirus* is primarily concentrated in the *P3* and *CI* gene regions [30,31,35]. Consequently, inserting introns into these two regions has become a standard strategy for clone construction. Gao et al. successfully developed stable infectious clonal vectors by inserting a single intron into the *P3* gene and dual introns into the *CI* gene of Tobacco vein banding mosaic virus (TVBMV). Reducing the number of introns led to viral sequence rearrangements and deletions, thereby confirming the essential role of these three intron insertion sites in maintaining clone stability [29]. Cheng et al. constructed infectious clones of PVY based on the strategy of Gao et al., further validating the effectiveness of multiple intron insertions [38]. By comparing various intron insertion patterns, Tuo et al. discovered that combined insertions in the *P3*-*CI* region significantly improved the stability of the full-length cDNA clone of Papaya leaf distortion mosaic virus (PLDMV) more effectively than modifications made to the single *CI* region. This enhancement led to increased assembly efficiency and a reduced risk of mutations and recombination [28].

In this study, infectious clones were constructed using seamless cloning and overlap PCR fusion intron methods. Based on prior experience, introns were preferentially inserted at the AG/GT splice site boundaries [32,39]. Comparative experiments involving single-site (*CI*-4240) and dual-site (*P3*-2955 and *CI*-4240) insertions revealed that while multiple intron insertions significantly enhanced clone stability, they could also lead to a loss of infectivity. Subsequently, based on the locations where mutations clustered after the insertion of a single intron, targeted insertions were performed at the *HC-Pro*-2431 and *CI*-4240 sites, ultimately achieving dual optimization of genetic stability and infectivity. Notably, unlike the previously reported unstable genomic regions of other *Potyvirus* species in *E. coli*, this study found that inserting an intron into the *P3* gene resolved the genetic stability issue in the host system but hindered the virus’s ability to complete gene expression and infection processes in tobacco hosts. Therefore, the position of intron insertion must comprehensively consider multiple factors, such as virus type and the number of introns.

## 4. Materials and Methods

### 4.1. Source of Virus

At the National Specialty Grain Crop Germplasm Resource Bank in Taiyuan, Shanxi Province, 20 legume samples suspected of being infected with BCMV were collected. These samples exhibited symptoms such as mosaic patterns, crinkling, and yellowing (Figure 1A). The collection included 8 common bean samples, 4 cowpea samples, 4 adzuki bean samples, and 4 mung bean samples. The field-collected samples were flash frozen in liquid nitrogen and transported to the laboratory, where they were stored in a −80 °C ultra-low temperature freezer for future analysis.

### 4.2. Small RNA Sequencing and Splicing

The collected four legume species were pooled into groups of four samples per group. Total RNA was extracted using the Trizol method (Biosharp, Beijing, China). The RNA samples were subsequently sent to Beijing Biomarker Technologies Co., Ltd. (Beijing, China) for small RNA sequencing. Following a quality assessment, libraries were constructed using the Small RNA Sample Prep Kit (Illumina, San Diego, CA, USA) and sequenced on the Illumina HiSeq platform for deep small RNA sequencing. Raw sequencing data were processed by removing sequences shorter than 18 nt or longer than 35 nt, as well as lacking the 3′ adapters and those in which the proportion of undetermined bases exceeded 10%. Clean reads were then filtered using Bowtie software (v1.0.0) to eliminate non-coding RNAs (ncRNAs), including ribosomal RNA (rRNA), transfer RNA (tRNA), small nuclear RNA (snRNA), and small nucleolar RNA (snoRNA), along with repetitive sequences. The remaining clean reads were assembled de novo using Velvet V1.0. The resulting contigs were analyzed for viral identification based on a prioritized annotation against viral databases. The presence of the predominant viral species identified was subsequently validated by reverse transcription-polymerase chain reaction (RT-PCR). The primer sequences are listed in Table S1. All primers used in this study were synthesized by Shanghai Sangon Biotech Co., Ltd. (Shanghai, China).

### 4.3. RT-PCR

Total RNA was extracted from leaf tissues using the Trizol method. Complementary DNA (cDNA) was then synthesized using the M-MLV Reverse Transcriptase First-Strand cDNA Synthesis Kit (Biomed, Beijing, China). PCR amplification was subsequently performed using a Taq DNA Polymerase Kit (Real-Times, Beijing, China). All procedures were conducted in strict accordance with the manufacturers’ instructions.

### 4.4. BCMV Whole Genome Sequencing

Referring to the sequencing methods described by Feng et al. [16,40,41], nine pairs of BCMV-specific primers were designed for the segmented amplification of the genomic RNA of the DY9 isolate. Each primer pair targets a specific nucleotide interval on the genome, yielding amplification fragments ranging from 900 to 2000 bp. The details are as follows: DY9-5′F/DY9-1R (1–1603 nt, 1603 bp), DY9-2F/DY9-2R (1016–2123 nt, 1108 bp), DY9-3F/DY9-3R (2015–2926 nt, 912 bp), DY9-4F/DY9-4R (2877–4239 nt, 1363 bp), DY9-5F/DY9-5R (4103–5557 nt, 1455 bp), DY9-6F/DY9-6R (4907–5961 nt, 1055 bp), DY9-7F/DY9-7R (5820–7228 nt, 1409 bp), DY9-8F/DY9-8R (7110–8911 nt, 1802 bp), and DY9-9F/DY9-3′R (8427–10,003 nt, 1577 bp), with overlapping regions incorporated between adjacent segments to facilitate subsequent sequence assembly. For terminal genomic sequences, the 3′-terminal sequence was obtained through reverse transcription (RT) using primer N1T, followed by PCR amplification with primers N1 and DY9-9F (Table S2). The 5′-terminal sequence was amplified using a 5′-RACE Kit (Accurate, Changsha, Changsha, China), and the resulting product was ligated into the pGEM^®^-T Easy Vector (Promega, Beijing, China). The target bands of the amplified or cloned products were sent to Shanghai Sangon Biotech Co., Ltd. (Shanghai, China) for sequencing. The obtained sequences were compared against those in the GenBank database using BLAST analysis. The complete viral genome was then assembled de novo using SeqMan Pro (Lasergene 7.1; DNASTAR, Madison, WI, USA). Open reading frames (ORFs) were predicted using ORF Finder (https://www.ncbi.nlm.nih.gov/orffinder/, accessed on 10 December 2023) and SnapGene software (v2.3.2). Ultimately, the complete genomic sequence of isolate DY9 was successfully obtained.

### 4.5. Nucleotide, Amino Acid and Phylogenetic Analysis

The nucleotide and amino acid sequence similarities of the DY9 sequence were compared with previously reported BCMV isolates in the NCBI database. Twenty full-length genomic sequences of BCMV isolates from various countries and regions were downloaded from the GenBank database. These sequences were aligned using the MUSCLE alignment function in MEGA 11 software. Subsequently, a phylogenetic tree was constructed using the Neighbor-Joining method to compare the DY9 sequence with other related BCMV isolates, and a phylogenetic analysis was performed. Bootstrap testing was conducted with 1000 replicates.

### 4.6. Pathogenic Differentiation

Eleven common bean cultivars with distinct genetic backgrounds were cultivated under standard growth conditions. When the first pair of true leaves had fully expanded (approximately 7–8 days after sowing), 0.5 g of wild-type BCMV-DY9-infected common bean leaf samples was collected and ground into a homogenate using a mortar and pestle with 5 mL of 1× PBS (leaf-to-buffer ratio of 1:10). Healthy bean plants treated solely with 1× PBS served as healthy controls. For inoculation, a small amount of carborundum powder was dusted onto the leaves of the eleven bean cultivars. Gloved hands were then dipped into the viral sap and used to gently rub the inoculated leaves back and forth. Following inoculation, the plants were maintained in a greenhouse under standard growth conditions (16 h photoperiod, day/night temperatures of 25/20 °C, and relative humidity of 60%) for subsequent analysis. Three weeks post-inoculation, the plants were subjected to RT-PCR detection and symptom observation to determine the pathotype of the wild-type BCMV-DY9 strain. Each cultivar included three biological replicates, and the pathotype assay was repeated independently three times.

### 4.7. Construction of pBCMV-DY9 Full-Length cDNA Clone

Based on the complete genomic sequence of BCMV-DY9, three cloned constructs were generated in this study for the purpose of constructing infectious clones. For the first construct, the viral genome was divided into two overlapping fragments for amplification: Fragment I (4477 bp, F: pDY9-5′F/R: DY9-4240R) and Fragment II (5798 bp, F: DY9-4240F/R: pDY9-3′R). Simultaneously, the full-length sequence of the binary vector pCB301 (7417 bp, F: pCB301-2F/R: pCB301-1R) was amplified. Fragment I was fused with Intron 1 (189 bp, derived from the *ST LS1* gene of *Solanum tuberosum*) using overlap PCR. The resulting fusion product, along with Fragment II and the linearized pCB301 vector, was seamlessly connected using the ClonExpress MultiS multi-fragment recombination system (Vazyme, Nanjing, China) to construct the clone pBCMV-DY9-1.

**Figure 5:**
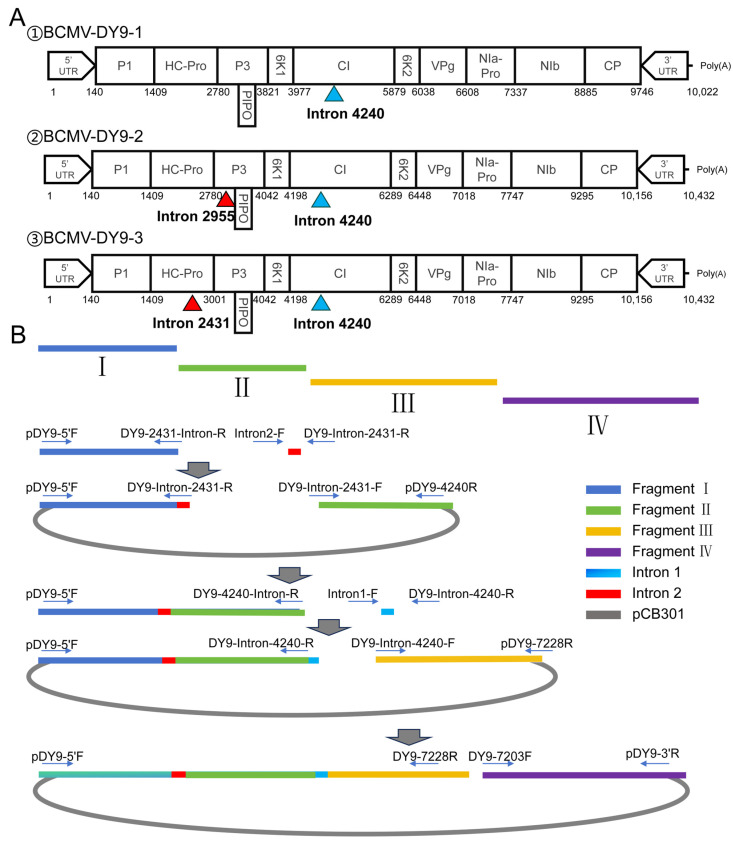
Construction strategy for an infectious full-length BCMV clone (pBCMV-DY9) with intron insertion. (**A**) Schematic diagram of the pBCMV-DY9 genome structure and the strategy for inserting introns at different positions. (**B**) Schematic overview of the stepwise assembly of the infectious BCMV clone (pBCMV-DY9-3) in this study.

The second structure employed a stepwise recombination strategy to divide the genome into four overlapping fragments: Fragment I (2991 bp, F: pDY9-5′F/R: DY9-2955-Intron-R), II (1305 bp, F: DY9-Intron-2955-F/R: DY9-4240R), III (2989 bp, F: DY9-Intron-4240-F/R: DY9-7228R), and IV (2834 bp, F: DY9-7203F/R: pDY9-3′R). First, Fragment I was fused with Intron 2 (221 bp, derived from the *NiR* gene of *P. vulgaris*) using overlap PCR. The resulting fusion product, Fragment II, was then ligated with linearized pCB301 using the ClonExpress MultiS recombination reaction system to construct the subclone pBCMV-DY9 A (4460 bp, I + Intron 2 + II). Subsequently, Intron 1 was fused with pBCMV-DY9 A (4649 bp) via overlap PCR. The fusion product (4649 bp), Fragment III, and linearized pCB301 were combined through homologous recombination to obtain the subclone pBCMV-DY9 B (7638 bp, I + Intron 2 + II+Intron 1 + III). Finally, Fragment IV was recombined with pBCMV-DY9 B (7638 bp) and linearized pCB301 through seamless cloning to obtain the full-length clone pBCMV-DY9-2 (10,432 bp, I + Intron 2 + II + Intron 1 + III + IV). A similar construction strategy was employed to generate pBCMV-DY9-3 (detailed procedures are illustrated in Figure 5). The primers used in the cloning construction described in this paper are listed in Table S3. In the experiments, PCR amplification was performed using the 2× HiFi Plus PCR Master Mix high-fidelity system (Biosharp, Beijing, China). The PCR products were purified with a universal DNA purification and recovery kit (Biosharp, Beijing, China). After measuring the concentration of the recovered products with a NanoDrop spectrophotometer, the quantities of multi-fragments and linearized vectors were calculated according to the instructions provided with the ClonExpress MultiS seamless cloning kit before being transformed into *E. coli* XL10-Gold competent cells (REBIO, Shanghai, China). All recombinant constructs were screened for positive clones following overnight incubation at 37 °C, and structural integrity was confirmed through full-length PCR amplification and sequencing.

### 4.8. Agrobacterium Inoculation

The recombinant plasmids were introduced into *A. tumefaciens* GV3101 competent cells using a Bio-Rad MicroPulser electroporator. Positive clones were screened and verified through colony PCR. Following culture expansion, an appropriate volume of bacterial suspension was inoculated into 20 mL of LB medium containing kanamycin resistance and rifampicin resistance, adjusting the initial OD_600_ nm to approximately 0.1. The bacterial cells were harvested when the culture was continuously shaken until the OD_600_ nm reached 0.4–0.6. These were then resuspended in a cell suspension buffer (100 µmol/L acetosyringone, 10 mmol/L MES [pH 5.6], 10 mmol/L MgCl_2_) to a final OD_600_ nm of 1.0 and incubated at room temperature in the dark for 2–3 h. *N. benthamiana* plants exhibiting robust growth with 4–6 true leaves were selected for inoculation. Vacuum infiltration was performed using a 1 mL needleless sterile syringe. Two to four true leaves per plant were infiltrated, with a total of ten independent plants inoculated. Plants infiltrated with *A. tumefaciens* carrying the empty pCB301 vector served as the negative control. All inoculated plants were subsequently maintained under standard growth conditions.

### 4.9. RT-qPCR Analysis

Samples of systemic leaves from *N. benthamiana* inoculated with the empty pCB301 vector, pBCMV-DY9, and wild-type virus DY9 were collected at 2 and 6 wpi for relative quantitative analysis of the virus. The samples were ground in liquid nitrogen, and total RNA was extracted. RNA concentration and purity were assessed using a NanoDrop spectrophotometer. First-strand cDNA was synthesized using the All-in-One First-Strand Synthesis Master Mix (with dsDNase) Kit (BestEnzymes, Nanjing, China). Real-time fluorescent quantitative PCR (qPCR) was conducted on the QuantStudio platform using Taq SYBR^®^ Green qPCR Premix (BestEnzymes, Nanjing, China). The *NtUBI* gene served as a reference gene for normalizing gene expression. The relevant primers are listed in Table S3. The relative expression level of the target gene in *N. benthamiana* samples was calculated using the 2^(−ΔΔCt)^ method. Experimental data were analyzed for significant differences using GraphPad Prism 9. Each treatment included three biological replicates, and the experiment was repeated three times.

### 4.10. Western Blot Analysis

A 0.1 g sample was ground into a fine powder in liquid nitrogen. Total protein was extracted by adding 150 μL of protein lysis buffer and 20 μL of β-mercaptoethanol, followed by vigorous vortexing for approximately 1 min. An appropriate volume of the protein sample was separated by SDS-PAGE (8% separating gel and a 5% stacking gel). Proteins were subsequently transferred from the gel onto a nitrocellulose (NC) membrane (Boster, Wuhan, China) using a wet transfer apparatus. Following the transfer, the NC membrane was blocked by shaking at 60 rpm for 2 h in 1× TBST buffer containing 5% skimmed milk powder. Immunoblotting was performed by incubating the membrane overnight at 4 °C with rotary agitation using a BCMV-CP specific antibody, R27 (kindly provided by Dr. Karasev, University of Idaho, Moscow, ID, USA), diluted to 1:15,000. The membrane was then transferred to a solution containing horseradish peroxidase (HRP)-conjugated goat anti-rabbit secondary antibody (diluted to 1:15,000; Sangon, Shanghai, China) and incubated with rotary agitation for 2 h. Finally, blot signals were visualized using an ECL chemiluminescence detection kit (Absin, Shanghai, China).

### 4.11. Transmission Electron Microscopy

Infected leaf tissue was added to pre-chilled (4 °C) 0.2 mol·L^−1^ PBS buffer (pH 7.2) containing 1% β-mercaptoethanol at a weight-to-volume ratio of 1:2 and ground into a homogenate. The homogenate was then filtered through sterile double-layered gauze. A 1:1 (*v*/*v*) mixture of 10% chloroform and n-butanol was added to the filtrate, followed by continued stirring in an ice bath for 15 min. The mixture was centrifuged at 10,000 rpm for 20 min at 4 °C. The supernatant was collected, supplemented with 4% sodium chloride and 4% polyethylene glycol (PEG 6000), and stirred on ice for 1.5 h. Subsequently, centrifugation was performed at 10,000 rpm for 15 min at 4 °C. The resulting pellet was retained, resuspended in 0.01 mol·L^−1^ PBS buffer (pH 7.2), transferred to a beaker, and stirred on ice for 1 h. The suspension was then centrifuged at 12,000 rpm for 20 min at 4 °C. The supernatant, containing the crude viral particle preparation (a milky-white viral suspension), was collected and stored at 4 °C.

The crude purified virus particles from pBCMV-DY9 were examined using a JEM-1400Flash transmission electron microscope (JEOL, Tokyo, Japan) with the negative staining method to visualize the virus particles of the infectious clone.

## 5. Conclusions

This study reports the first successful construction and confirmation of infectivity for an infectious clone of a PG-III strain of BCMV. The development of this viral infectious clone establishes a foundation for investigating the functions of the BCMV genome and its pathogenic mechanisms. Furthermore, it provides new insights and strategies for constructing infectious clones of other potyviruses or larger-genome RNA viruses.

## Figures and Tables

**Table 1:** Summary of small RNA sequencing results.

Sample	Sequencing Data Statistics Table					
	Raw Reads	GC (%)	Q30 (%)	Length <18	Length >35	Clean Reads
S1	11,033,010	50.21	96.13	748,214	268,695	10,016,101
S2	17,506,053	50.51	96.86	631,051	1,133,097	15,741,905
S3	22,810,943	50.07	96.46	924,370	527,897	21,358,676
S4	24,377,556	50.05	96.78	730,897	1,853,403	21,793,256
S5	20,799,009	50.29	96.87	552,737	1,148,189	19,098,083

**Table 2:** Sequence assembly annotation results for small RNA sequencing of five field-mixed samples.

Sample	Virus Species	Reference Virus Sequences	Number of Contigs	Contig Length (nt)	Coverage /(%)	Similarity /(%)
S1	*Bean common mosaic virus* (BCMV)	NC_003397.1	402	20~163	76	87.93~100
	*Cucumber mosaic virus* (CMV)-1	NC_002034.1	46	17~232	56	90.29~100
	*Cucumber mosaic virus* (CMV)-2	NC_002035.1	22	26~350	73	89.09~100
	*Cucumber mosaic virus* (CMV)-3	NC_001440.1	24	17~196	41	90.36~100
	*Bean yellow mosaic virus* (BYMV)	NC_003492.1	0	—	0	0
	*Soybean mosaic virus* (SMV)	NC_002634.1	1	40	3	90.37~100
	*Cowpea aphid-borne mosaic virus* (CABMV)	NC_004013.1	2	30~50	5	94.21~100
S2	*Bean common mosaic virus* (BCMV)	NC_003397.1	289	17~220	85	83.33~100
	*Cucumber mosaic virus* (CMV)-1	NC_002034.1	23	17~366	54	86.36~100
	*Cucumber mosaic virus* (CMV)-2	NC_002035.1	27	41~372	80	86.34~96.88
	*Cucumber mosaic virus* (CMV)-3	NC_001440.1	36	17~191	67	89.63~100
	*Bean yellow mosaic virus* (BYMV)	NC_003492.1	42	54~675	40	86.87~98.95
	*Soybean mosaic virus* (SMV)	NC_002634.1	2	35~60	8	87.14~98.77
	*Cowpea aphid-borne mosaic virus* (CABMV)	NC_004013.1	2	27~57	7	85.78~98.35
S3	*Bean common mosaic virus* (BCMV)	NC_003397.1	148	17~114	78	89.80~100
	*Cucumber mosaic virus* (CMV)-1	NC_002034.1	32	17~242	75	87.95~100
	*Cucumber mosaic virus* (CMV)-2	NC_002035.1	32	17~437	79	84.78~100
	*Cucumber mosaic virus* (CMV)-3	NC_001440.1	44	41~214	72	91.80~100
	*Bean yellow mosaic virus* (BYMV)	NC_003492.1	0	—	0	0
	*Soybean mosaic virus* (SMV)	NC_002634.1	72	41~378	34	86.34~100
	*Cowpea aphid-borne mosaic virus* (CABMV)	NC_004013.1	1	38	3	88.32~99.56
S4	*Bean common mosaic virus* (BCMV)	NC_003397.1	139	17~198	60	88.64~100
	*Cucumber mosaic virus* (CMV)-1	NC_002034.1	88	17~206	43	92.52~100
	*Cucumber mosaic virus* (CMV)-2	NC_002035.1	34	33~313	52	91.09~100
	*Cucumber mosaic virus* (CMV)-3	NC_001440.1	40	17~116	32	93.69~100
	*Bean yellow mosaic virus* (BYMV)	NC_003492.1	0	—	0	0
	*Soybean mosaic virus* (SMV)	NC_002634.1	1	254	4	75.38
	*Cowpea aphid-borne mosaic virus* (CABMV)	NC_004013.1	32	185~678	33	76.90~89.45
S5	*Bean common mosaic virus* (BCMV)	NC_003397.1	157	17~178	79	88.89~100
	*Cucumber mosaic virus* (CMV)-1	NC_002034.1	39	34~278	73	85.57~97.83
	*Cucumber mosaic virus* (CMV)-2	NC_002035.1	34	17~371	65	85.78~100
	*Cucumber mosaic virus* (CMV)-3	NC_001440.1	24	23~128	44	88.76~100
	*Bean yellow mosaic virus* (BYMV)	NC_003492.1	0	—	0	0
	*Soybean mosaic virus* (SMV)	NC_002634.1	1	77	2	90.34~97.21
	*Cowpea aphid-borne mosaic virus* (CABMV)	NC_004013.1	0	—	0	0

**Table 3:** Sequential homology of the complete genome and *CP* gene of BCMV-DY9 relative to other BCMV and BCMNV isolates.

GenBank	Isolate Name	Country	Total Sequence Identity (%)	*CP* Gene Sequence Identity (%)
OR778613.1	BCMV-22Huhe	China	91.61	93.38
MF405191.1	BCMV-HXH-2	Tanzania	90.56	93.96
LC582403.1	BCMV-HY	China	90.57	94.77
AY112735.1	BCMV-NL1	England	90.46	93.38
KY057338.1	BCMV-NL7n	India	90.39	93.73
MH024839.1	BCMV-Viva2	USA	90.42	93.96
MW620827.1	BCMV-NE15-44	Nepal	90.42	93.73
MG640408.2	BCMV-INIFAP CJ30	Mexico	90.36	94.19
MW620826.1	BCMV-NE15-43	Nepal	90.40	93.61
MH220847.1	BCMV-424	Australia	90.37	94.08
KU896809.1	BCMV-2 Iowa	USA	90.36	93.96
MG640401.2	BCMV-INIFAP CG28	Mexico	90.34	93.73
MG640402.2	BCMV-INIFAP CG32	Mexico	90.34	93.96
MG640406.2	BCMV-INIFAP CJ20-6	Mexico	90.30	94.08
MH024841.1	BCMV-3PF	USA	90.48	93.96
KM023744.1	BCMV-NL1	USA	90.38	93.96
KF114860.1	BCMV-NL-1n	India	90.29	94.19
MH220846.1	BCMV-425	Australia	90.33	94.08
MG640410.2	BCMV-INIFAP CN22	Mexico	90.32	93.84
MG640413.2	BCMV-INIFAP CN61	Mexico	90.29	93.84
HQ229995.1	BCMNV-TN1	Tanzania	77.89	78.41
AY282577.1	BCMNV-NL-3	Netherlands	79.66	80.65
HQ229993.1	BCMNV-NL5	Netherlands	79.52	80.48
HQ229994.1	BCMNV-NL8	Netherlands	77.98	78.61
AY864314	BCMNV-NL-3K	USA	79.66	80.65
NC_004047	BCMNV-NL-3	Netherlands	79.38	80.31

**Table 4:** List of BCMV Pathogenic Types and Identification of DY9 Pathogenic Strains.

Bean Cultivar	Resistance Genes	Pathogroup								DY9
		PG- I	PG- II	PG- III	PG- IV	PG- V	PG- VI	PG- VII	PG- VIII	PG- III
Dubbele Witte	None	+	+	+	+	+	+	+	+	+
SGR	i,bc-u	+	+	+	+	+	+	+	+	+
RGLC	i,bc-u,bc-1		+		+	+	+	+		
RGLB	i,bc-u,bc-1^2^				+		+	+		
Sanilac	i,bc-u,bc-2			+		+	+		+	+
UI35	i,bc-u,bc-1^2^, bc-2^2^							+		
IVT7214	i,bc-u,bc-2, bc-3								+	
Jubila	I,bc-1									
Amanda	I,bc-1^2^									
US1006	I,bc-u,bc-2^2^									
IVT7233	I,bc-u,bc-1^2^, bc-2^2^									

Note: The bean varieties consisted of 11 types containing different combinations of resistance genes. Based on whether BCMV strains could infect different bean varieties, the strains were classified into pathogenicity groups PG-I to PG-VIII. A “+” indicates that bean varieties can be infected by strains within that pathogenicity group.

## Data Availability

The small RNA sequencing data have been deposited at the NCBI Sequence Read Archive under BioProject accession PRJNA1311737.

## Supplementary Materials

The following supporting information can be downloaded at: https://www.mdpi.com/article/10.3390/plants14213359/s1, Additional File S1: Table S1. Virus-specific primers used in this study. Table S2. Primer sequence information for BCMV-DY9 whole genome amplification. Table S3. pBCMV-DY9 infectious clone construction primers. Additional File S2: Complete nucleotide sequence and map of the BCMV-DY9 cDNA and pBCMV-DY9 constructs described in this paper.

## Abbreviations

The following abbreviations are used in this manuscript:

BCMNV	Bean common mosaic necrosis virus
BCMV	Bean common mosaic virus
bp	Base pair
BYMV	Bean yellow mosaic virus
CABMV	Cowpea aphid-borne mosaic virus
cDNA	Complementary DNA
CMV	Cucumber mosaic virus
E. Coli	Escherichia coli
HPLC	Reversed-phase high-pressure liquid chromatography
ICTV	International committee on taxonomy of viruses
kDa	Kilodalton
ncRNAs	Non-coding RNAs
nt	Nucleotide
ORF	Open reading frame
PLDMV	Papaya leaf distortion mosaic virus
PVY	Potato virus Y
RACE	Rapid amplification of cDNA ends
RT-PCR	Reverse transcription-polymerase chain reaction
SMV	Soybean mosaic virus
TVBMV	Tobacco vein banding mosaic virus
UTRs	Untranslated regions
wpi	Weeks post-inoculation
